# Rupture of an unsuspected ectopic pregnancy following a hysterosalpingography—A case report

**DOI:** 10.1016/j.ijscr.2019.01.015

**Published:** 2019-01-29

**Authors:** P.T. Lim, Rohit, V. Viardot-Foucault

**Affiliations:** a Department of Obstetrics and Gynaecology, KK Women’s and Children’s Hospital, Singapore; b Department of Diagnostic Radiology, KK Women’s and Children’s Hospital, Singapore; c Department of Reproductive Medicine, KK Women’s and Children’s Hospital, Singapore

**Keywords:** Case report, Hysterosalpingography, Ectopic pregnancy, Subfertility

## Abstract

**Introduction:**

Hysterosalpingography (HSG) is routinely performed in the mid-follicular phase of a woman’s menstrual cycle for cavity and tubal patency assessment as a part of the infertility screening. A pre-procedural pregnancy test is not routinely required unless the patient reports abnormal menstrual bleeding or irregular menstrual pattern.

**Presentation of case:**

We present the case of a 29 year-old sub fertile woman who had a HSG performed on day 12 of her menstrual cycle. She developed abdominal pain shortly after and was diagnosed with a ruptured ectopic pregnancy, resulting in a right salpingectomy. The patient was discharged well 3 days later.

**Discussion:**

Based on available guidelines and the rare occurrence of such complication, we find insufficient evidence to perform universal pre-procedural pregnancy testing. However, we strongly recommend doing it for abnormal menstrual cycles (cycles shorter than 21 or longer than 35 days, unreliable menstrual history or unusual menstrual flow pattern). Furthermore, HSG should be scheduled during the follicular phase after practicing safe sex within the two weeks preceding the test.

**Conclusion:**

While pre-procedural pregnancy test should not be performed for all, a high index of suspicion for early pregnancy should be maintained as undetected ectopic and heterotopic pregnancies could lead to serious complications once the HSG is done.

## Highlights

• HSG should be scheduled during the follicular phase, between Day 5 and Day 12 of a 28 days menstrual cycle and only after menstrual flow has stopped.
• Patients should practice safe sex during the cycle where HSG is performed.
• Patients should be informed about the risk of undetected pregnancy that could lead to serious complications once the HSG is done.
• Women with cycles < 21 or >35 days, with unreliable menstrual history or unusual menstrual flow pattern, should have a pregnancy test before HSG.
• At present, there is insufficient evidence to recommend universal pre-procedural pregnancy testing.

## 1 Introduction

Hysterosalpingography (HSG) is a minimally invasive radiographic imaging of the uterine cavity and fallopian tubes involving the injection of contrast media with fluoroscopic visualization. It is often used as the first line of assessment in a context of subfertility, commonly performed within the first 5–12 days of the menstrual cycle after the cessation of menstrual flow [1]. A pre-procedural pregnancy test is not routinely required unless the patient reports abnormal menstrual bleeding or irregular menstrual pattern [1]. The incidence of performing HSG in an undetected pregnancy is low hence there is limited data available on the long-term adverse pregnancy outcomes related to the exposure to ionizing radiation or the mechanical trauma to the embryo. We present a case of a patient managed in a tertiary public hospital for whom a HSG was performed while an unsuspected ectopic pregnancy was ongoing, leading to a rupture. We will then discuss the role of routine pre-procedural pregnancy test for patients undergoing HSG.

This case report has been written in line with the SCARE criteria [2].

## 2 Presentation of case

A 29 year-old Chinese woman, gravida 2 para 1, consulted a public tertiary hospital after 8 months of secondary subfertility despite regular sexual intercourse. She had no significant past medical history or family history. She was not on any long term medications. Her clinical examination was normal with a body mass index of 24.9 kg/m^2^. Her menstrual cycles were longer than usual but regular (36–40 days with 5 days of menstrual flow).

**Fig. 1:**
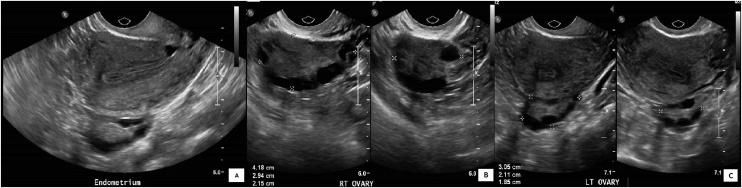
Transvaginal ultrasound pelvis images. [A] Sagittal view of the uterus with normal endometrium thickness [B] Right ovary enlarged with polycystic morphology (vol14cc). [C] Left ovary is normal.

**Fig. 2:**
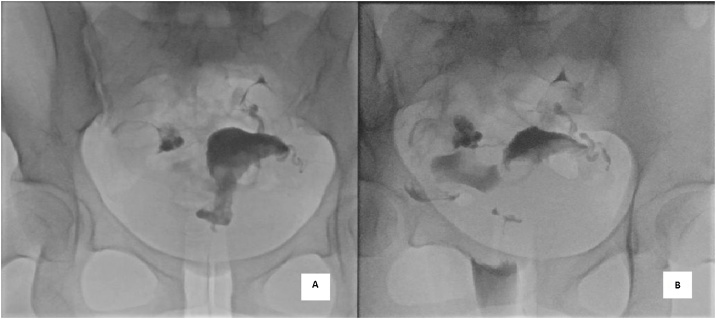
Hysterosalpingography [A] Smooth triangular uterine cavity filled with contrast [B] dilated right fallopian tube, filling with contrast but with presence of free spillage.

Hormonal investigations revealed normal gonadotropins and ovulatory cycles (Table 1). The baseline transvaginal ultrasound of the pelvis showed a normal uterus, and a right polycystic ovarian morphology (volume 14cc) (Fig. 1). The semen analysis was consistent with an isolated teratozoospermia. She reported a normal menstrual flow starting on 7th November 2016 and was scheduled for an HSG on 18^th^ November 2016 (day 12 of menstrual cycle). The menstrual period prior to this was on 30th September 2016. No pregnancy test was done prior to the HSG because the reported menstrual flow was normal and on time. The HSG was reported as normal, with both fallopian tubes being opacified and showing free intraperitoneal spillage (Fig. 2).

**Table 1:** Hormonal investigations revealed normal gonadotropins and ovulatory cycles.

Investigation	Units	Reference Range	12/08/2016	27/10/2016
Follicle-stimulating hormone	IU/L	1.35–17.06	4.9	
Luteinizing Hormone	IU/L	0.38–60.33	22.85	
Progesterone	nmol/L	Follicular phase: 2.03–14.1 Mid Luteal phase: 19.1–79.5	5.69	53.69 (Day 29)
Estradiol	pmol/L	77–2382	362	
Testosterone	nmol/L	0.5-1.9	1.6	
Prolactin	mcg/L	7.0–32.9	5.3	
Anti-Mullerian Hormone	ng/ml	4.0–6.8	6.3	
Thyroid Stimulating Hormone	mIU/L	0.50–4.50	–	0.9
Free Thyroxine	pmol/l	10.3–25.7	–	14

A few hours after the HSG, she developed intermittent lower abdominal pain that progressively exacerbated. She consulted the emergency department on 22nd November 2016. She was tachycardic with a heart rate of 106 beats/min and blood pressure was stable at 105/69 mmHg. She was given fluid resuscitation and was monitored in the critical care area in the emergency department. On examination, there was tenderness over the right iliac fossa and suprapubic region with guarding but no rebound tenderness. Vaginal examination revealed tenderness over the right adnexa and blood at the cervix. The urine pregnancy test was found to be positive with a serum beta HCG level of 29,271 mIU/mL. A bedside transvaginal ultrasound showed an empty uterus with a thickened endometrium and free fluid in the pouch of Douglas. Her hemoglobin level was 11.2 g/dL. Renal function and coagulation profile were normal. The diagnosis of a ruptured ectopic pregnancy was suspected and confirmed by laparoscopy when a therapeutic right salpingectomy was performed by a gynecology specialist. Intra-operatively, there was a ruptured right tubal ectopic pregnancy approximately 4 cm in size and hemoperitoneum with an estimated blood loss of 600mls. Her post-operative recovery was unremarkable and she was discharged 3 days later. She was well when reviewed in the outpatient clinic 3 months later and did not report any post-operative complication.

## 3 Discussion

This is a case of an undetected ectopic pregnancy that ruptured after a HSG was performed. To date, there have been very few reports of this kind. A review of 6225 HSG by Justesen et al found an incidence of 4 cases (0.06%) of inadvertently performed examinations during early pregnancy [3]. He reported a case of a 34 year-old woman whose last vaginal bleeding started a few days before the expected time and lasted only for one day. She reported this as a menstrual bleeding, but an intrauterine pregnancy was found at HSG. She was initially treated as per threatened abortion as she presented with lower abdominal pain and bloody vaginal discharge 5 days after HSG. 4 weeks later, she was readmitted and a laparotomy revealed a ruptured right ectopic pregnancy [3].

In another case series by Cheung et al., a 23 year old woman with regular menstrual cycles underwent a HSG five days after her reported menstrual period [4]. The radiology report showed no abnormalities and tubes were patent. A week later, she presented with lower abdominal pain and bleeding that was assumed as being part of an endometritis and was treated with antibiotics. She was then readmitted for increasing lower abdominal pain a week later and the diagnosis of a right ectopic pregnancy was suspected (urine pregnancy test positive, empty uterus with a right adnexal mass on pelvic ultrasound). A diagnostic laparoscopy was performed confirming the presence of a right tubal ectopic pregnancy and a right salpingectomy was performed.

Cheung et al also reported two other cases – one with a spontaneous abortion and another with an intrauterine pregnancy which resulted in a healthy live birth at 39 weeks gestation. The child was followed-up and was reported to have normal growth and development at 7 years of age [5].

While our patient reported a “normal menstrual flow”, it is not uncommon to have bleeding from a decidualized cycle which may be easily mistaken as a menstrual flow [6]. Moreover, her history of long menstrual cycles and scan findings of unilateral polycystic ovarian morphology are suggestive of polycystic ovarian syndrome even though one of her cycle was proven to be ovulatory (adequate luteal phase progesterone level). Indeed, a normal menstrual cycle length is defined as being between 21 to 35 days and the extension of her cycles’ length beyond 35 days could have been considered as abnormal and a pre-procedural pregnancy test done. The positivity of the test would have allowed early detection and treatment of this ectopic pregnancy and therefore a possible preservation of her full fertility potential. The early treatment of an ectopic pregnancy is often conservative and consists of either medical treatment with Methotrexate or surgery with salpingotomy instead of salpingectomy. The overall success rate of methotrexate for tubal ectopic pregnancy was quoted as 65–95% with 3–27% of women requiring a second dose [7]. In patients with fertility – reducing factors such as previous pelvic inflammatory disease, it is found that there are higher rates of subsequent intrauterine pregnancy in patients with salpingotomy performed rather than salpingectomy [7]. In this case, though methotrexate would be unsuitable in view of the high beta HCG level, a salpingotomy, rather than a salpingectomy could have been performed if the ectopic pregnancy was detected earlier and the rupture probably avoided.

In order to avoid performing a HSG during early pregnancy, the American College of Radiology advocates that HSG should be scheduled between day 7–10 of the menstrual cycle and a pregnancy test should be done if there is any suspicion of pregnancy [1]. Similarly, the American College of Obstetricians and Gynecologists does not advocate a routine pre-procedural pregnancy test, and advise performing HSG within the first 14 days of the menstrual cycle. In our institution, HSG is scheduled between Day 5–12 of the menstrual cycle, and only after cessation of menstrual flow. Patients are also advised to avoid sexual intercourse during that menstrual cycle. They are required to fill in a pre-procedural questionnaire which includes questions about their menstrual cycle pattern, abnormal bleeding or irregular menstruation. A pregnancy test is performed if there is a positive answer.

As we review the literature for cases of undiagnosed pregnancies which have undergone HSG, we raise the question of whether a pre-procedural pregnancy test should be adopted for all.

In a prospective study performed by Herr et al, which implemented a mandatory point-of-care urine pregnancy testing before HSG, only one out of 410 (0.025%) women were found to have unsuspected early pregnancy [8]. In our institution where approximately 1800 HSGs were performed in 2016, only one case was performed during an unsuspected pregnancy. Upon reviewing our hospital data, no other case was reported previously.

Most of the studies focused on the cost-effectiveness of routine pre-procedural pregnancy test to prevent an extremely rare complication. However, due to the severe complications that can possibly arise from a misdiagnosis, the cost of the pregnancy test is not the only financial component to consider but also the additional cost derived from unnecessary hospitalization, surgical procedure and treatment of iatrogenic infertility.

While there is insufficient evidence at the moment to support the use of a universal mandatory pregnancy tests prior to HSG, we propose an individualized approach whereby clinicians would perform a pregnancy test for selected women based on the following stricter criteria defining abnormal menstrual cycles: cycle length shorter than 21 days or longer than 35 days; unusual menstrual flow pattern and unreliable menstrual history. A menstrual diary may improve the accuracy of self-reported symptoms and also assist from a medico legal standpoint.

## 4 Conclusion

In conclusion, we concur with international guidelines that there is at present, insufficient evidence to recommend performing a pre-procedural pregnancy test for all. However, we should always maintain a high index of suspicion for early pregnancy in general as undetected ectopic and heterotopic pregnancies could lead to serious complications once the HSG is done.

This can be improved by scheduling the HSG during the follicular phase and advising patient to practice safe-sex during the cycle when the test has been planned. A pre-procedural pregnancy test should be performed for women with irregular cycles and for women with unreliable menstrual history or unusual menstrual flow pattern.

## Conflict of interest

There is no conflict of interest to disclose.

## Sources of funding

No funding is required for this manuscript.

## Ethical approval

This study is exempt from ethical approval in our institution.

## Consent

Written informed consent was obtained from the patient for publication of this case report and accompanying images. A copy of the written consent is available for review by the Editor-in-Chief of this journal on request.

## Author contribution

Dr PT Lim is the corresponding author who is also the main writer of the manuscript. Dr Veronique is the specialist in reproductive medicine involved in the care of the patient during her outpatient clinic visits. She was also the main author who conceptualised, supervised and edited this case report. Dr Rohit is the radiologist who reported the hysterosalpingogram and was also involved in editing of the case report.

## Registration of research studies

UIN: Researchregistry4439.

## Guarantor

Dr Veronique Celine Viardot-Foucault.

Senior Consultant.

KK Women’s and Children’s Hospital.

Department of Reproductive Medicine.

## Provenance and peer review

Not commissioned, externally peer-reviewed.

## Additional information

There is no conflict of interest to disclose and there is no funding required for this study. This study is exempt from ethical approval in our institution.
